# Bispecific CAR-T cells targeting both CD19 and CD22 for therapy of adults with relapsed or refractory B cell acute lymphoblastic leukemia

**DOI:** 10.1186/s13045-020-00856-8

**Published:** 2020-04-03

**Authors:** Hanren Dai, Zhiqiang Wu, Hejin Jia, Chuan Tong, Yelei Guo, Dongdong Ti, Xiao Han, Yang Liu, Wenying Zhang, Chunmeng Wang, Yajing Zhang, Meixia Chen, Qingming Yang, Yao Wang, Weidong Han

**Affiliations:** 1 Department of Molecular Biology and Immunology, Institute of Basic Medicine, Chinese PLA General Hospital, No. 28 Fuxing Road, Beijing, 100853 China; 2 Department of Bio-therapeutic, Chinese PLA General Hospital, No. 28 Fuxing Road, Beijing, 100853 China; 3 State Key Laboratory of Pharmaceutical Biotechnology, Nanjing University, Nanjing, China; 4 Department of Geriatric Hematology, Chinese PLA General Hospital, Beijing, China

**Keywords:** Bispecific, CAR, CD19, CD22, B-ALL, Immunotherapy, Tumor antigen escape

## Abstract

**Background:**

Despite the impressive complete remission (CR) induced by CD19 CAR-T cell therapy in B-ALL, the high rate of complete responses is sometimes limited by the emergence of CD19-negative leukemia. Bispecific CAR-modified T cells targeting both CD19 and CD22 may overcome the limitation of CD19-negative relapse.

**Methods:**

We here report the design of a bispecific CAR simultaneous targeting of CD19 and CD22. We performed a phase 1 trial of bispecific CAR T cell therapy in patients with relapsed/refractory precursor B-ALL at a dose that ranged from 1.7 × 10^6^ to 3 × 10^6^ CAR T cells per kilogram of body weight.

**Results:**

We demonstrate bispecific CD19/CD22 CAR T cells could trigger robust cytolytic activity against target cells. MRD-negative CR was achieved in 6 out of 6 enrolled patients. Autologous CD19/CD22 CAR T cells proliferated in vivo and were detected in the blood, bone marrow, and cerebrospinal fluid. No neurotoxicity occurred in any of the 6 patients treated. Of note, one patient had a relapse with blast cells that no longer expressed CD19 and exhibited diminished CD22 site density approximately 5 months after treatment.

**Conclusion:**

In brief, autologous CD19/CD22 CAR T cell therapy is feasible and safe and mediates potent anti-leukemic activity in patients with relapsed/refractory B-ALL. Furthermore, the emergence of target antigen loss and expression downregulation highlights the critical need to anticipate antigen escape. Our study demonstrates the reliability of bispecific CD19/CD22 CAR T cell therapy in inducing remission in adult patients with relapsed/refractory B-ALL.

**Trial registration:**

ClinicalTrials.gov identifier: NCT03185494.

## Background

Two anti-CD19 chimeric antigen receptor (CAR) T cell (CD19 CAR T cell) products, tisagenlecleucel and axicabtagene, were the first US Food and Drug Administration (FDA)-approved gene-modified cell therapies for any indication [1, 2]. Despite the great successes with CAR T cell therapy in leukemia that have been published previously by our group and others [3–7], up to 60% of relapses after CD19 CAR T cell therapy are characterized by CD19 antigen loss, which involves several different mechanisms [8–10]. Hence, the development of improvements in CAR design to target antigen loss represents a critical need. One approach to overcoming antigen loss following CAR T cell therapy is to simultaneously target more than one antigen on cancer cells, an approach that is compelling for B-ALL, given that anti-CD22 CAR T cells have also demonstrated clinical efficacy [11].

Some studies have indicated that compared with single-antigen targeting, dual-antigen or multiantigen targeting by CARs may result in synergistic responses in solid tumors, optimizing response rates to therapy [12, 13] and preventing antigen escape [14, 15]. Preclinical data supporting the multitargeted approaches include tandem anti-CD19-CD20 CAR constructs; combinatorial anti-CD19 and anti-CD123 strategies, targeting both CD19 and CD22; and several clinical trials utilizing strategies are underway.

Here, we report the results of using autologous CD19/CD22 CAR T cells in six adult patients with chemotherapy-resistant or refractory ALL. All patients went into remission with no minimal residual disease (MRD), showing the efficacy of the bispecific CD19/CD22 CAR T cells, and remission was accompanied by robust expansion of the CAR T cells in vivo in these patients who were previously considered to have refractory and incurable disease.

## Methods

### Clinical protocol design

We conducted open-label, phase 1 clinical trials (ClinicalTrials.gov number, NCT03185494) at the Chinese PLA general hospital that was designed to assess the safety and feasibility of autologous CD19/CD22 CAR T cell therapy in adult patients with relapsed/refractory B-ALL. Eligible patients with B-ALL had relapsed disease or disease refractory to standard therapy plus at least one salvage regimen. The eligibility criteria included the presence of measurable disease, an adequate performance status, and sufficient organ function. The study protocols were approved by the institutional review board at the Chinese PLA general hospital, and the patients provided written informed consent. This clinical investigation was conducted according to the principles of the Declaration of Helsinki. There was no commercial support for this study. Conditioning chemotherapy regimens aimed at depleting endogenous leukocytes that can inhibit the anti-malignancy activity of adoptively transferred T cells [16–18] were chosen by the investigator on the basis of each patient’s treatment history, blood cell counts, and organ function (Table S1) and were administered 1 week before infusion of autologous bispecific CD19/CD22 CAR T cells. The CD19/CD22 CAR T cells were infused 1 day after the completion of the conditioning chemotherapy regimen. These six patients included in the study were given fludarabine (30 mg/m^2^ per day) on days 4, 3, and 2 and cyclophosphamide (30 mg/kg per day) on days 2 and 1. The cells were infused over 30 min on day 1. The dose ranged from 1.7 × 10^6^ to 3 × 10^6^ CAR T cells per kilogram of body weight. Additional experimental details are included in the Supplementary Material.

Response assessment was performed 1 month after CD19/CD22 CAR T cell infusion. Complete remission (CR) was defined as less than 5% bone marrow (BM) blasts, the absence of circulating blasts, and no extramedullary sites of disease, regardless of cell count recovery. Complete response with incomplete count recovery was a complete response with cytopenia. MRD negativity was defined as less than 0.01% bone marrow blasts by flow cytometry [19, 20]. Relapsed disease was defined as the reappearance of blasts in the blood or bone marrow or in an extramedullary site after a complete remission.

### Generation of CD19/CD22 CAR T cells

Peripheral blood mononuclear cells (PBMC) were collected from the enrolled patients by leukapheresis. The leukapheresis products were washed and cryopreserved. T cells from thawed leukapheresis products were isolated and activated with Dynabeads Human T-Activator CD3/CD28 magnetic beads. PBMCs were cultured in X-VIVO 15 medium supplemented with interleukin (IL)-2. Two days after the initial T cell activation, the T cells were transduced with a lentivirus encoding the CD19/CD22-BB-z transgene. The cells were expanded for an additional 10 days. Quality checks were performed during the CAR T cell manufacturing process.

### Assessment of CD19/CD22 CAR T cell persistence

The persistence of bispecific CD19/CD22 CAR T cells in patient peripheral blood, BM, and cerebrospinal fluid (CSF) was assessed by fluorescent-activated cell sorting (FACS) and quantitative PCR (qPCR) as described in the Supplementary Material.

### Soluble factor analysis

Serial serum samples obtained before and after cell infusion were analyzed as described in the supplementary material.

### CD22 flow cytometric site density determination by flow cytometry

Refer to the Supplementary Material.

## Results

### In vitro validation of bispecific CD19/CD22 CAR T cells

**Fig. 1:**
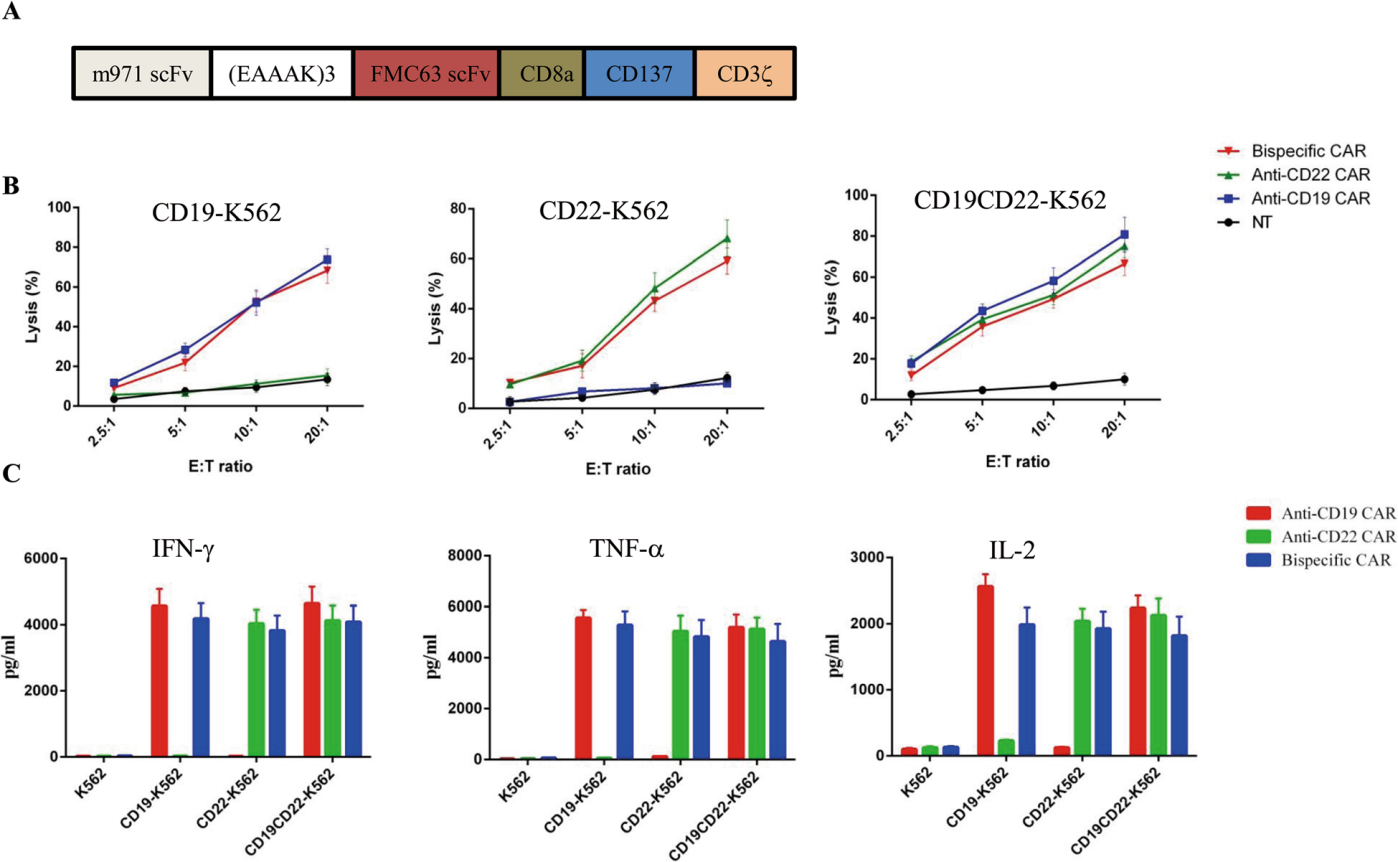
Preclinical evaluation of bispecific CD19/CD22 CAR T cells. **a** Schematic of the bispecific CD19/CD22 CAR structure. **b** Cytotoxicity of CAR T cells against target cells carrying luciferase reporter gene evaluated by luminescent assay, after co-culturing with tumor cells for 4 h at the indicated E:T ratios, with CAR T cells. **c** Cytokine production by anti-CD19 CAR-, anti-CD22 CAR-, and bispecific CAR-expressing T cells co-incubated with K562, K562-CD19, K562-CD22, and K562-CD19CD22 cell lines. Bars represent mean + SD of replicate samples. Data are representative of three independent experiments performed with CAR T cells from three separate donors. A two-tailed, unpaired two-sample *t* test was used for statistical analysis

Second-generation CD19/CD22 CARs were constructed by taking the standard three-domain CAR architecture and incorporating two single-chain variable fragments (scFvs) connected in tandem via an EAAAK linker (Fig. 1a). The heavy and light chains of m971 were linked by a (GGGS)_3_ sequence. The generated CD19/CD22 CAR T cells were extensively evaluated in a series of in vitro immunological assays. The cytotoxicity of the CD19/CD22 CAR T cells was further evaluated, and the cells demonstrated activity comparable to that of CD19 and CD22 monovalent CAR T cells (Fig. 1b). We found that T cells transduced with the CD19/CD22 CAR vector yielded strong induction of cytokines in the presence of tumor target that is similar in magnitude (Fig. 1c).

### Clinical responses

Tumor burdens varied among the enrolled patients at the time of T cell infusion (Table 1). Of the six treated subjects, five patients had a higher disease burden, with 5% or more BM blasts. One patient (patient 5) had minimal residual disease with 3.66% bone marrow blasts. Following bispecific CD19/CD22 CAR T cell therapy, all patients experienced MRD-negative CR as assessed by FACS.

**Table 1:** Patient characteristics and response summary

Patient no.	Age	Sex	Cytogenetics at diagnosis	Previous treatment	Number of relapses	Marrow blasts (% of mononuclear)		CAR dose (10^6^/kg)	Response (day 30)	Months until relapse after CAR	CRS	Neurological toxicity
						Pre-treatment (%)	Post-CAR (%)					
1	23	M	Normal karyotype	C	2	39.94	< 0.01	3	CR, MRD Neg	10	Grade 1	None
2	24	F	Normal karyotype	C	2	55.27	< 0.01	2	CR, MRD Neg	5	Grade 1	None
3	44	F	Normal karyotype	C	3	5.81	< 0.01	2	CR, MRD Neg	11+	Grade 1	None
4	17	M	Normal karyotype	C	1	54	< 0.01	3	CR, MRD Neg	3	Grade 1	None
5	20	M	Complex including 12p13 deletion	C, R	2	3.66	< 0.01	2	CR, MRD Neg	8+	Grade 2	None
6^#^	39	M	Ph^+^	C	2	89.14	< 0.01	1.7	CR,MRD Neg	8+	Grade 2	None

*M* male, *F* female, *C* chemotherapy, *R* radiation therapy, *CR* complete remission, *CRS* cytokine release syndrome, *MRD* minimal residual disease

^#^Patient 6 received allo-hematopoietic stem cell transplant 2 months after CD19/CD22 CAR T cell therapy

**Fig. 2:**
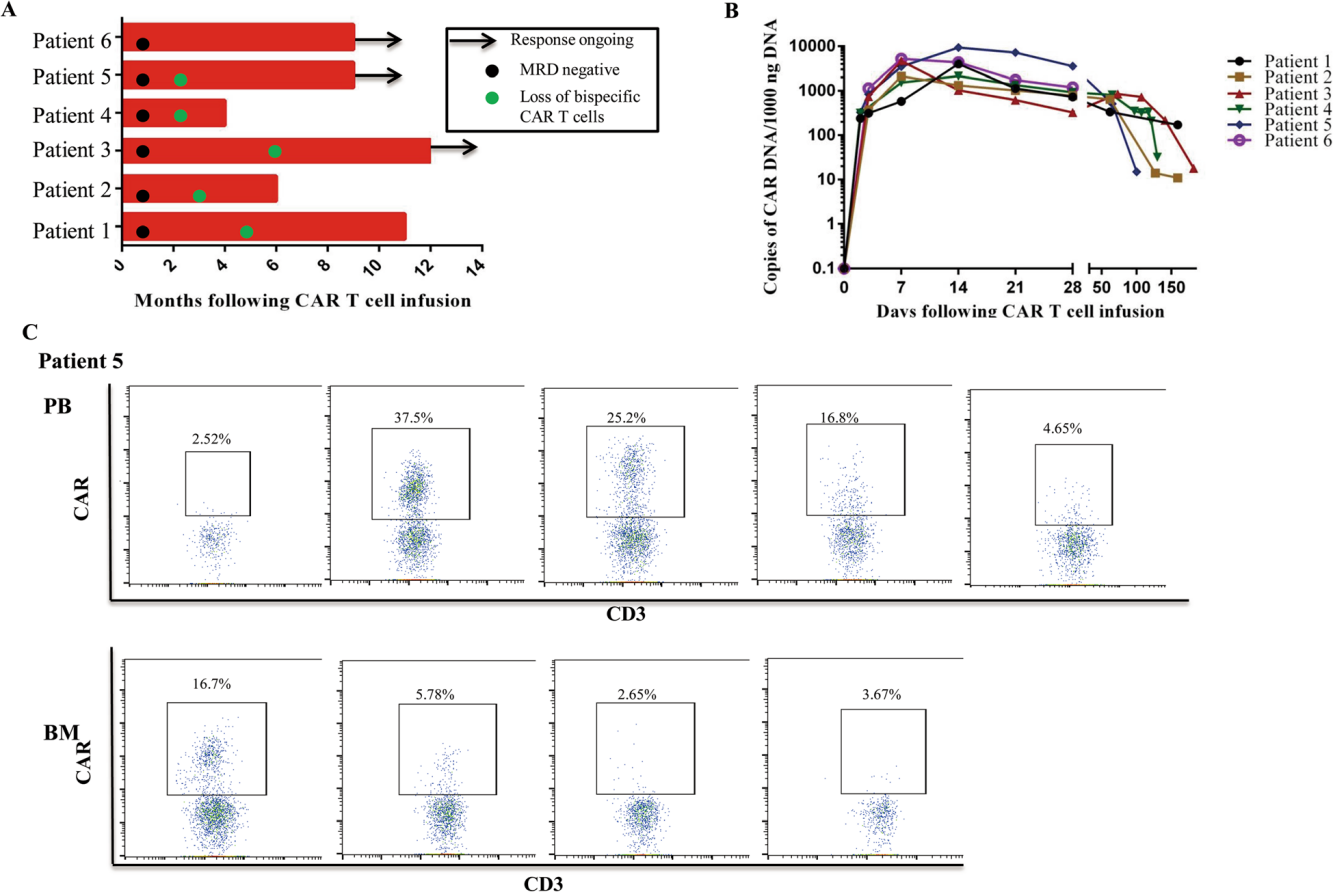
Expansion and persistence of bispecific CAR T cells and tumor response in patients treated with bispecific CAR T cells. **a** Treatment response of each patient after bispecific CAR T cell treatment and the duration of response. Ongoing remission is marked by a black arrow. Patient number is shown to the left. **b** The presence of CD19/CD22 CAR T cells in the peripheral blood as assessed by quantitative real-time polymerase chain reaction (PCR) assay. Genomic DNA was isolated from samples of whole blood samples collected at serial time points before and after cell infusion. of the *y* axis.. **c** The results of flow cytometry analysis showing in vivo expansion of bispecific CAR T cells in the peripheral blood and bone marrow of representative patient 5, who achieved a MRD negative. Both the *x* and *y* axes are log10 scales. MRD, minimal residual disease

Patient 3 was in a morphologic CR with a molecular remission at the first assessment 1 month after infusion (Fig. 2a). Her BM at 1, 2, and 6 months after the cell infusions showed sustained absence of the blasts (Fig. S1). She remained in MRD-negative CR (11 months) at the time of publication.

After treatment on the CD19/CD22 CAR T cell protocol, patient 5 achieved MRD-negative CR 1 month after therapy (Fig. 2a). Additionally, patient 5, in whom blast cells were detected in the CSF at the time of infusion, subsequently had complete clearance of CNS leukemia at his most recent follow-up, and no CNS relapses were observed (Fig. S2). His CR had been sustained for more than 8 months at the time of this report. Patient 6 achieved an MRD-negative status on day 30 (Fig. 2a) and remained in MRD-negative CR up to the time of allo-HSCT 2 months after therapy. Three patients, specifically patient 1, patient 2, and patient 4, relapsed at 10 months, 5 months, and 3 months after cell therapy, respectively. Despite these cases of relapsed disease, the presented clinical outcomes for this cohort of patients collectively demonstrate the profound clinical benefit of CD19/CD22 CAR T cell therapy in the setting of relapsed adult B-ALL, a highly aggressive and predominantly fatal condition [21, 22].

### Bispecific CD19/CD22 CAR T cell expansion, systemic inflammatory markers, and B cell aplasia

Bispecific CD19/CD22 CAR T cells were detected in the peripheral blood, BM, and CSF by both flow cytometry and qPCR. More than a 3-log peak expansion of CAR T cells was noted in vivo by 2 weeks after infusion (Fig. 2b). The frequency of CAR T cells in the circulation progressively increased in vivo to 47.1% of total T cells in patient 1, 12.8% in patient 2, 16.8% in patient 3, 15% in patient 4, 37.5% in patient 5, and 32.6% in patient 6 (Fig. S3). CSF specimens adequate for analysis were collected from the six patients and exhibited detectable CSF-infiltrating CD19/CD22 CAR T cells (Fig. S3 and S4). Patient 5 had evidence of CNS leukemia at the time of cell infusion that disappeared coincident with a rise in CSF-infiltrating CD19/CD22 CAR T cell numbers (Fig. 2c and S4). We observed high persistence of CD19/CD22 CAR T cells in the last assessment of patient 3 (Fig. S3). Flow cytometric analysis of samples from both the blood and the BM 6 months after infusion revealed the presence of CAR T cells as well as the absence of B cells in patient 3 (Figs. S1, S3 and S4). The CAR T cells persisted in all six patients beyond 3 months, as shown by flow cytometric and qPCR analyses (Figs. 4b and S3). This is in contrast to the other studies reported by groups at the University of Pennsylvania [5, 23], who reported CAR T cell persistence for several months to even more than a year.

**Fig. 3:**
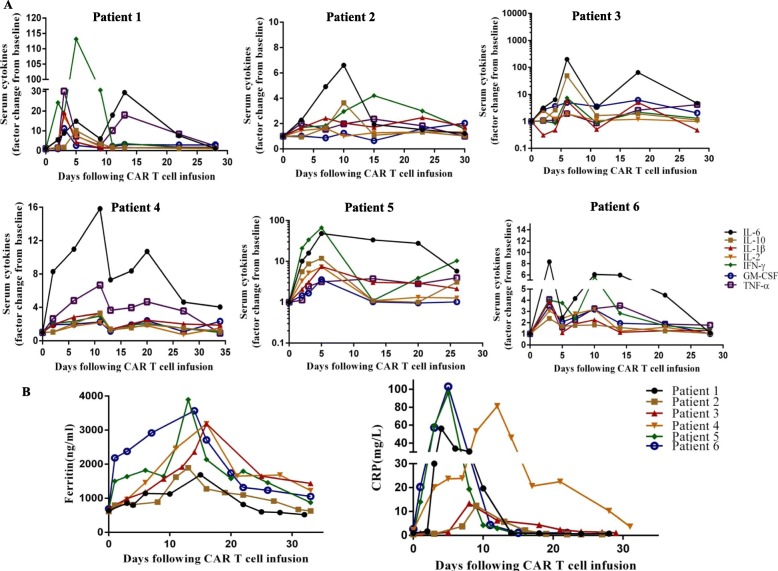
Serum cytokines, inflammatory markers, and B cell markers before and after bispecific CD19/CD22 CAR T cell therapy. **a** Serum levels of cytokines and inflammatory markers measured at the indicated time points after cell infusion. Analytes with a fold change greater than or equal to three are indicated and plotted as relative changes from the baseline (determined on day 0 before infusion). Increases in the levels of cytokines, including interleukin-1β, GM-CSF, interleukin-2, interleukin-6, and interleukin-10, tumor necrosis factor α, and interferon gamma, were observed in the three patients. **b** Serum ferritin and C-reactive protein concentrations. **c** Circulating blasts and low levels of nonmalignant B cells in patient 5 peripheral blood at day 1, followed by CAR T cell expansion coincident with the clearance of leukemia and nonmalignant B cells by day 20; the CD19/CD22 CAR T cells disappeared and B cell recovery occurred by day 100. Green dots show leukemic blasts (CD19+CD34+); red dots represent normal B cells

Among the six patients, transient elevations in the levels of cytokines in the serum occurred during CRS after infusion; elevations in interleukin-6, interleukin-10, and ferritin levels were prominent. Similar tendencies were observed in the levels of other cytokines, including interferon gamma, GM-CSF, interleukin-1β, interleukin-2, and tumor necrosis factor α (Fig. 3a, b). We also observed a transient increase in the C-reactive protein level, but with this parameter exhibited substantial variability (Fig. 3b). These observed patterns are similar to the patterns observed previously in patients with ALL who experienced leukemia remission after CD19 CAR T cell infusion [1].

We used flow cytometry to detect B cells to assess patients for the development of B cell aplasia in the patients. B cell aplasia in the BM and blood was observed in all patients within 1 month after infusion. In patient 5, circulating leukemia cells were present on day 1, and dramatic CAR T cell expansion was observed on day 14, consistent with the elimination of normal B cells and malignant blasts, followed by the disappearance of CD19/CD22 CAR T cells by day 100 with the reconstitution of normal B cells but continued absence of blasts (Fig. 3c).

### Toxicity of CD19/CD22 CAR T cells

**Fig. 4:**
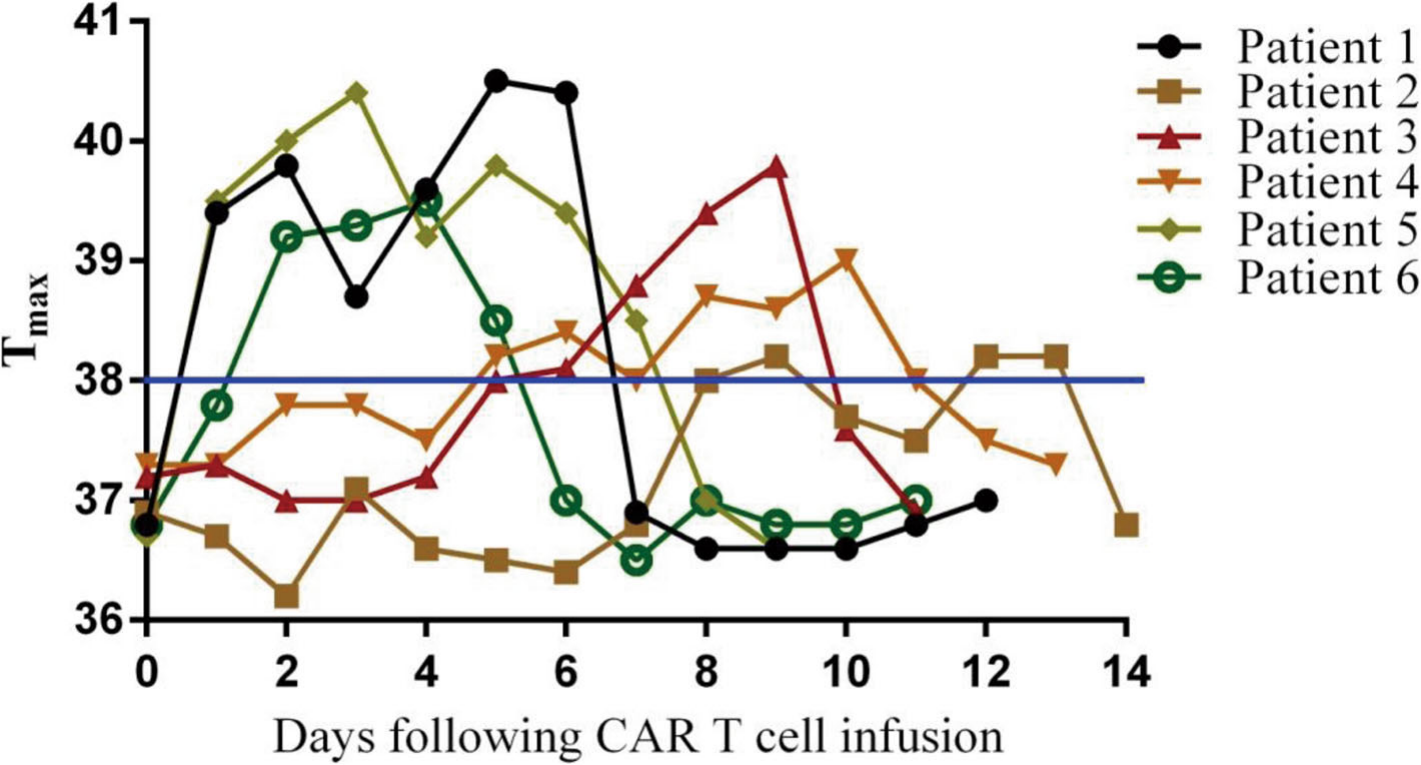
Persistent fevers in patients with acute lymphoblastic leukemia after infusion with bispecific CAR T cells. Changes in body temperature after cell infusion, including the maximum temperature per 24-h period in the six patients. The green line marks the minimum temperature for a fever (38 °C)

Adverse events of special interest that may be related to bispecific CD19/CD22 CAR T cell therapy are summarized in Table 1. All toxicities resolved to normal or baseline levels. The severity of cytokine release syndrome (CRS) was graded according to consensus criteria [24, 25]. Neurological toxicities were graded according to immune effector cell-associated neurotoxicity syndrome (ICANS) [26]. No grade 3 or 4 CRS was observed in any of the six treated patients. Patient 1 experienced fever on day 2, which subsequently resolved on day 7 after cell infusion. Patient 2 experienced low-grade fevers for 5 days beginning on day 8 after infusion (Fig. 4). Patient 3 experienced fever on day 5, and subsequent defervescence occurred on day 10 (Fig. 2). Patient 4 experienced fever beginning 6 days after cell therapy. The fever persisted for 7 days and resolved. Patient 5 developed a febrile syndrome and hypotension, with rigors beginning 2 days after infusion (Fig. 4). The fevers persisted for approximately 7 days and resolved after administration of tocilizumab; he had no further constitutional symptoms. Patient 6 experienced fever and hypotension. The fevers persisted for approximately 4 days and resolved after administration of tocilizumab. In addition, all patients had anemia, decreased white blood cell counts, and decreased platelet counts that resolved. For all patients, the CSF showed evidence of CNS trafficking by the infused CD19/CD22 CAR T cells, and the concentrations of CSF-infiltrating CAR T cells were highest in patient 5 with CNS leukemia (Fig. S3); however, no neurotoxicity was observed in any of the treated patients.

### Relapse after cell therapy

**Fig. 5:**
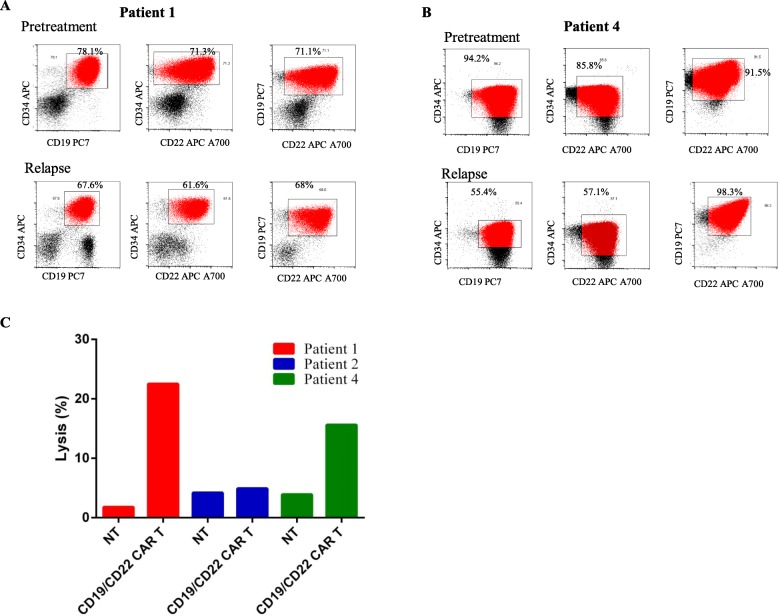
Changes in CD19 and CD22 cell surface expression in relapsed patient baseline and at the time of relapse. Bone marrow samples were obtained from patients 1 and 2 before cell infusion and at the time of relapse. Mononuclear cells isolated from the bone marrow samples were stained for CD45, CD34, CD19, and CD22. After gating on live cells, the blast gate (CD45+side scatter [SSC] low) was subgated on CD34^+^ cells, and histograms were generated for CD19 and CD22 expression. **a** CD19 and CD22 expression of B-ALL tumor cells in patient 1 from the initial diagnostic BM sample and the relapsed sample. **b** CD19 and CD22 expression of B-ALL tumor cells in patient 4 from the initial diagnostic BM sample and the relapsed sample. **c** Non-transduced (NT) T cells from the leukapheresis product or CAR T cells from the end-of-product formulated cells were incubated with the relapsed B-ALL tumor cells from patients 1, 2, and 4. Effectors were incubated with tumor cells at a 50:1 effector/target ratio for 4 h. Supernatants were harvested and analyzed using the CytoTox 96 Non-Radioactive Cytotoxicity Assay kit (Promega). **d** Changes in the CD19 cell surface expression in patient 2 between the baseline time point and the time of relapse. **e** Direct Sanger sequencing performed with patient 2 cDNA. Mutations in exon 2 of CD19 were found in the relapse samples from patient 2 and were predicted to result in a truncated protein. **f** Emergence of leukemia cell populations with CD22 expression different from that of the cells harvested before treatment. **g** The change in CD22 cell surface expression in patients 1, 2, and 4

Patients 1 and 4 relapsed at 10 months and 3 months after CD19/CD22 CAR T cell therapy, respectively. The relapsed leukemia cells expressed the target CD19 and CD22 antigens at pretreatment levels (Fig. 5a, b). Furthermore, the recurrent leukemia cells remained sensitive to lysis by autologous CD19/CD22 CAR T cells (Fig. 5c). The relapses observed in these two patients, who previously achieved MRD-negative CR, were therefore not due to antigen escape but may be imputed to be, at least in part, to the result of the abrogated persistence of the infused CD19/CD22 CAR T cells, which suggests a loss of efficacy CD19/CD22 CAR T cell-mediated surveillance of leukemia.

Flow cytometric analysis revealed that patient 2 experienced relapse at 5 months after CD19/CD22 CAR T cell therapy with CD19-negative leukemia cells and low but variable CD22 surface expression on the leukemic blasts. Using patient 2 cDNA as a template, we observed a deletion in exon 2 of CD19, which accounted for the CD19 loss observed (Fig. 5d, e and Table S1), but no acquired mutations within the CD22 locus. Diminished CD22 site density in the leukemic blasts, as determined by flow cytometry, was adequate to evade the infused CAR T cells (Fig. 5f, g), thus enabling leukemia relapse, and these results were consistent with those of the other clinical studies [11]. In addition, the total CD22 mRNA level was slightly increased at that time that the CD22 site density was diminished (Fig. S5). Briefly, patient 2 relapsed after cell therapy through an acquired tumor resistance mechanism (loss of CD19 and diminished expression of CD22 to a level below the threshold needed to elicit anti-tumor functions).

## Discussion

Adult patients with relapsed B-ALL have a dismal outcome with current therapies, especially patients who relapse within 6 months of their CR1. After the CR1, which is relatively easy to achieve, it becomes very difficult to achieve a second complete remission (CR2) when the patient relapses again. In adult patients, CR2 rates are lower than 50% [27].

Despite unprecedented successes in early phase trials of anti-CD19 CAR T cells for the treatment of relapsed/refractory B-ALL, relapses with CD19 loss occurs in a large fraction of patients [28]. Some studies [29, 30] have indicated that simultaneous targeting of multiple antigens may reduce the likelihood of single target loss-induced relapse, similar to the well-established paradigm of multiagent combination chemotherapy regimens. Here, we report that autologous bispecific CAR T cells targeting both CD19 and CD22 mediated MRD-negative CRs in six adult patients with recurrent B-ALL.

FACS analysis of bone marrow from the three patients with disease relapse following bispecific CD19/CD22 CAR T cell therapy indicated two major patterns: relapse of antigen-positive tumor cells and relapse associated with antigen loss. ALL antigen-positive relapse is often associated with limited CAR T cell persistence [5]. Patients 1 and 4 had loss of CAR T cell persistence at the time of relapse. Notably, in vitro experiments indicate that relapsed leukemia cells retain CD19 expression and sensitivity to bispecific CD19/CD22 CAR T cells ex vivo. These results suggest that relapse with CD19+ cells represents a potential opportunity for additional infusions of CAR T cells; however, repeated treatment strategies to treat CD19+ relapse occurring with the loss of CAR T cell persistence have unfortunately produced limited responses. Some groups have reported poor outcomes following reinfusion into patients with B cell non-Hodgkin lymphoma, but the use of an intensified lymphodepletion regimen containing fludarabine in addition to cyclophosphamide improves the reinfusion response and enhances initial CAR T cell expansion and persistence [31]. This concept of intensified lymphodepletion has similarly been used in reinfusion strategies for other CAR-based therapies and has produced improved clinical outcomes [32]. In addition, recipient anti-CAR immune responses that might lead to the loss of CAR T cell have been demonstrated against CAR T cells in clinical trials [18, 33]. A solution to potentially reduce immunogenicity of CAR binding domains is to use human instead of murine sequences.

Although some studies [34] have indicated that dual targeting is more effective in preventing antigen escape than sequential anti-CD19 CAR T cell and anti-CD22 CAR T cell infusions, we observed the unfortunate emergence of both CD19^−^ and CD22^dim^ blast cells in one patient treated with bispecific CD19/CD22 CAR T cells on this protocol. Thus far, established mechanisms leading to loss of CD19 expression include alternative splicing [8], acquired mutations [9], and lineage switch [10]. Additionally, trogocytosis, whereby CAR T cells themselves acquire CD19 on the cell surface leading to both a reduction in the target antigen level on the tumor cell surface and more importantly CAR T cell death via fratricide, may lead to diminished responses or disease relapse [34]. In contrast to the relapsing mechanism in most resistant leukemias that occurs after CD19 CAR T cell therapy, relapse after CD22 CAR T cell therapy typically occurs with sustained, but diminished, cell surface CD22 expression on leukemia cells [11], thus implicating post-transcriptional mechanisms in this biology. The emergence of CD19^−^ and CD22^dim^ escape variants has been reported in patients sequentially infused with anti-CD19 CAR T cells and anti-CD22 CAR T cells [11, 35]. It is possible that coinfusing CAR T cells targeting CD123 or CD38, in addition to those targeting CD19 and CD22, may diminish the likelihood of this event. Some studies indicated that sequential infusion of CAR19/22 T cell is a promising approach to reduce antigen loss relapse in CD19/CD22-directed therapy [36]. In brief, this case provides proof-of-concept data on the potential for target antigen loss in leukemia following bispecific targeted immunotherapy and, within the growing field of novel targeted immunotherapies, highlights the importance of tumor evaluation throughout the course of treatment. We propose that reengineering CARs for multispecificity may diminish the likelihood of tumor escape through antigen loss. Proof of principle for the bioactivity of a bispecific CAR T cells in humans is demonstrated here.

For adult patients who achieve CR after CAR T cell therapy, treatment choices are very limited. Allo-HSCT may be a reasonable option. However, it is controversial whether allo-HSCT improves the outcomes of patients who achieve MRD-negative CR after CAR T cell therapy. A previous study showed that no significant differences in event-free survival or OS were found between patients who underwent transplantation and those who did not [20]. However, Pan et al. reported that CAR T cell therapy bridging to allo-HSCT could significantly improve the clinical outcomes of refractory/relapsed B-ALL patients [37]. In our study, similar to CD19 CAR T cell therapy, bispecific CD19/CD22 CAR T cell therapy provided a temporal window for patients otherwise ineligible or eligible under very suboptimal conditions (MRD+) to bridge to potentially life-saving allo-HSCT.

The primary CD19/CD22 CAR T cell-attributable toxicity observed was one instance of grade 1 and 2 CRS, which was similar to macrophage activation syndrome [5, 38, 39]. Hyperferritinemia is a characteristic of these conditions. We observed substantial elevations in the ferritin level during the first month after infusion in our patients. In addition, we observed no evidence of neurotoxic effects, even in patient 5 with CNS leukemia, which is consistent with a previous report that observed follow-up therapy with anti-CD22 CAR T cells [11]. The adverse events of CRS and neurotoxicity are closely related to marked increases in the serum levels of cytokines produced directly by rapidly proliferating CAR T cells after encountering CD19+ tumor cells or normal B cells or indirectly by myeloid cells activated by the CAR T cells [40–43]. In contrast to previous studies showing marked increases in serum cytokine concentrations after CD19 CAR T cell therapy [1, 3–5], our study found that serum cytokine and immune modulator levels were moderately increased after cell infusion in all treated patients. The results of in vitro and in vivo studies indicate that incorporating both CD19 and CD22 scFv sequences is critical for cytokine production in CAR T cells. Several studies have suggested important roles for the scFv framework region sequences in CAR T cell activation and function [29, 30]. This study provides an avenue for CAR design with additional scFv sequences to improve the safety and efficacy of CAR T cell therapy.

The patterns of expansion and persistence of CD19/CD22 CAR T cells with a 4-1BB costimulatory endodomain are similar to those observed for anti-CD19 CAR T cells incorporating the 4-1BB endodomain [4, 5]. CD19/CD22 CAR T cells also migrated efficiently to the CSF, raising the prospect that CD19/CD22 CAR T cells can prevent or treat CNS leukemia. Our results show the efficacy of CD19/CD22 CAR T cells in adults with acute leukemia.

## Conclusions

The clinical responses of patients receiving a bispecific CD19/CD22 CAR T cell infusion have prompted the expansion of our phase 1 study to determine the success rate, durability, and toxicity events associated with this treatment in a larger group of patients. The results in these patients with relapsed/refractory B-ALL suggest that direct bispecific CD19/CD22 CAR T cells may be necessary to preempt antigen escape without exacerbating toxicity.

## Supplementary information

**Additional file 1.**

## Data Availability

Not applicable.

## Abbreviations

Allo-HSCT: Allo-hematopoietic stem-cell transplant
B-ALL: B cell acute lymphoblastic leukemia
BM: Bone marrow
CAR: Chimeric antigen receptor
CNS: Central nervous system
CR: Complete remission
CR2: Second complete remission
CRS: Cytokine release syndrome
FDA: Food and Drug Administration
IL-2: Interleukin-2
MRD: Minimal residual disease
PBMC: Peripheral blood mononuclear cells
scFv: Single-chain variable fragments
