# Correction to: Bispecific CAR-T cells targeting both CD19 and CD22 for therapy of adults with relapsed or refractory B cell acute lymphoblastic leukemia

**DOI:** 10.1186/s13045-020-00878-2

**Published:** 2020-05-18

**Authors:** Hanren Dai, Zhiqiang Wu, Hejin Jia, Chuan Tong, Yelei Guo, Dongdong Ti, Xiao Han, Yang Liu, Wenying Zhang, Chunmeng Wang, Yajing Zhang, Meixia Chen, Qingming Yang, Yao Wang, Weidong Han

**Affiliations:** 1 Department of Molecular Biology and Immunology, Institute of Basic Medicine, Chinese PLA General Hospital, No. 28 Fuxing Road, Beijing, 100853 China; 2 Department of Bio-therapeutic, Chinese PLA General Hospital, No. 28 Fuxing Road, Beijing, 100853 China; 3 State Key Laboratory of Pharmaceutical Biotechnology, Nanjing University, Nanjing, China; 4 Department of Geriatric Hematology, Chinese PLA General Hospital, Beijing, China

**Correction to: J Hematol Oncol 13, 30 (2020)**

**https://doi.org/10.1186/s13045-020-00856-8**

The original article [1] contained omissions in Fig. 2, Fig. 3 & Fig. 5 which were mistakenly introduced by the production team handling this article. The original article has now been corrected to reflect the correct presentation of these figures.
