# Supplementary material for: Bispecific CAR-T cells targeting both CD19 and CD22 for therapy of adults with relapsed or refractory B cell acute lymphoblastic leukemia
Source: J Hematol Oncol. 2020 Apr 3;13:30. doi: 10.1186/s13045-020-00856-8 (PMC7126394; doi:10.1186/s13045-020-00856-8)
Supplement: Supplementary file 1 — Additional file 1. [file 13045_2020_856_MOESM1_ESM.docx]

**Contents**

Methods………………………………………………………………..…2

Generation of bi-specific CD19/CD22 CAR T cells………………….…2

CAR detection ……………………………………………………….. …2

Q-PCR analysis…………………………………………………………..3

Soluble factor analysis…………………………………………….……...4

Flow cytometry………………………………………………….……..…4

CD22 flow cytometric site density determination…………………..……5

Patient clinical background………………………………………………6

**Generation of bispecific CD19/CD22 CAR T cells**

**A** bispecific CD19/CD22 CAR recombinant lentiviral vector was designed and referred to as pRRL-EF1A-19/22CAR. It included the following components from 5′ to 3′: the VSVG lentiviral backbone, the FMC63 scFv, the m971 scFv, the hinge and transmembrane regions of the CD8 molecule, the cytoplasmic portions of 4-1BB, and the cytoplasmic component of the CD3-ζ molecule. The vector was modified with the EF1A promoter. Peripheral blood mononuclear cells (PBMCs) were collected by leukapheresis, and T cells were enriched by mononuclear cell elutriation, washed, and expanded by stimulation with anti-CD3/CD28–coated paramagnetic beads for T cell activation. The lentiviral vector containing the previously described CD19-CD22-BB-ζ transgene was added at the time of cell activation and was washed out on day 3 after culture initiation. The next day, the cells were returned to culture in AIM V medium with 5% human AB serum and 300 international units/mL IL-2. Cells were infused into patients on day 10 of culture. The final product release criteria in the IND included the following: cell viability ≥ 80%, CD3+ cells ≥ 90%, residual paramagnetic anti-CD3/CD28-coated paramagnetic beads ≤ 100 per 3x10^6^ cells, endotoxin ≤ 3.5 EU/mL, Mycoplasma negative, negative bacterial and fungal cultures, residual bovine serum albumin ≤ 1 μg/mL, VSV-G DNA (as a surrogate marker for replication-competent lentivirus) ≤ 50 copies per μg of DNA, and transduction efficiency measured by flow cytometry ≥ 5%.

**CAR detection**

For each T cell culture and freshly thawed PBMC sample that was analyzed, the cells were stained with biotinylated polyclonal goat anti-mouse-F(ab)_2_ antibodies (anti-Fab, Jackson ImmunoResearch) to detect the antiCD19 scFv. The cells were then stained with phycoerythrin (PE)-labeled streptavidin (BD) and an anti-CD3 antibody.^1^ Surface expression of the CD22 CAR on transduced T cells was determined by flow cytometry using a CD22-Fc chimera (R&D Systems), followed by incubation with an antigen-presenting cell (APC)-F(ab)2 specific for human immunoglobulin G (IgG)-Fc (Jackson ImmunoResearch Laboratories).

**qPCR to quantitate blood CAR T cells**

For each patient, DNA was extracted from PBMCs collected before treatment and at multiple time points after treatment. DNA was extracted by using a Qiagen DNeasy blood and tissue kit. DNA from each time point was amplified in duplicate with a primer and probe set (Applied Biosystems) that was specific for the CAR. Real-time PCR was carried out with a Roche Light Cycler 480 real-time PCR system. An 8-point standard curve was generated consisting of 10^6^ to 5 copies of the lentivirus plasmid spiked into 100 ng of nontransduced control genomic DNA. Each data point (samples, standard curve, and reference samples) was evaluated in triplicate with average values reported. The primers for the CAR transgene were described previously.^2^ A parallel amplification reaction to control for the quality of interrogated DNA was performed using 12-20 ng of input genomic DNA, a primer/probe combination specific for a nontranscribed genomic sequence upstream of the CDKN1A gene (GENEBANK: Z85996) (sense primer: 5’- GAAAGCTGACTGCCCCTATTTG-3’, antisense primer: 5’-GAGAGGAAGTGCTGGGAACAAT-3’), and an 8-point standard curve created by dilution of control genomic DNA; these amplification reactions produced a correction factor (CF) (ng detected/ng input). Copies of transgene/microgram of DNA was calculated according to the following formula: copies calculated from the standard curve/input DNA (ng) × CF × 1000 ng. The accuracy of this assay was determined by the ability to quantify marking of the infused cell product by qPCR.^3^

**Soluble factor analysis**

Quantification of soluble cytokine factors was performed using an Aimplex Premixed Analyte kit according to the manufacturer’s protocol (YSL Bioprocess Development Co). The assay is based on the principle of the sandwich ELISA. Briefly, each bead in a population is conjugated with capture antibodies specific for one cytokine. This antibody traps the protein of interest in a sample. The amount of the analyte captured is detected via a biotinylated antibody against a secondary epitope in the protein, followed by streptavidin-R-phycoerythrin (PE) treatment. The concentrations of cytokines in samples are determined by comparing the fluorescence signals of the samples against a standard curve generated from serial dilutions of known concentrations of the analyte. The assay protocol consists of a 60-min incubation step to allow antigen capture by [antibody-conjugated](https://www.sciencedirect.com/topics/immunology-and-microbiology/antibody-conjugate) beads, a 30-min incubation step for biotinylated-antibody detection of the analyte and a 20-min streptavidin-PE incubation step. The fluorescence intensities of the beads are measured using a flow cytometer. In this study, each sample was tested in duplicate, and the mean of two samples was used for analysis. The data were processed using [FlowJo](https://www.sciencedirect.com/topics/immunology-and-microbiology/data-analysis-software) software, and each cytokine concentration is expressed in pg/mL.^4^

**Flow cytometry**

Multiparametric immunophenotyping was performed using approximately 4×10^6^ total cells/condition, using fluorescence minus one (FMO) stains as described in the text. The cells were then washed once with cold PBA, resuspended in 0.5% formalin and stored at 4°C for up to 12 h before acquisition. Approximately 2-3 million cells were acquired for each staining cocktail. Compensation values were established using single-antibody stains and BD compensation beads (Becton Dickinson) and were calculated and applied automatically by the instrument software. Data were analyzed using FlowJo software (Version 10, TreeStar).

**CD22 site density determination by flow cytometry**

The antigen site density on patient blasts was quantified by determining the anti-CD22 antibody binding capacity per cell using a BD Bioscience QuantiBRITE system for fluorescence measurements.^5^ In brief, cell-surface antigen expression was evaluated by determining the ABC (antibodies bound per cell, the mean value of the maximum capacity of each cell to bind a monoclonal antibody) of lymphoblasts for an anti-CD22 antibody (clone S-HCL-1, BDIS) using saturating concentrations of the antibody and a BD Bioscience QuantiBRITE system (QuantiBRITE standard beads and QuantiCALC software) for fluorescence quantification. QuantiBRITE beads were acquired on a flow cytometer on the same day at the same instrument settings used for the individual specimens. A standard curve comparing the geometric mean of fluorescence to the known PE content of the QuantiBRITE beads was constructed using QuantiCALC software (BD Bioscience). The regression analysis, slope, intercept, and correlation coefficient were determined. Analysis gates were drawn based on CD10, CD19, and CD45 copositivity to include only the malignant cells for determination of the geometric mean fluorescence of CD22 staining. ABC values were generated from the measured geometric mean fluorescence of only the malignant cells using the QuantiBRITE standard curve.

**Patient clinical background**

Six patients with relapsed B-ALL comprised the first cohort of patients in this clinical trial and were treated by infusion of the lowest planned dose of CD19/CD22 CAR T cells (1.7× 10^6^ to 3.0 × 10^6^ CAR+ T cells/kg) (Table 1, Table S1 and S3). The patients all had progressive malignancy at the time of enrollment in our protocol.

Patient 1 was first diagnosed with B-ALL with normal cytogenetics in 2017 at age 21 when he presented with fatigue and leukocytosis. He achieved his first complete remission (CR1), which persisted throughout VDCLP chemotherapy, but his cancer recurred 4 months later, and he did not have a response to further intensive chemotherapy regimens, including treatments with cyclophosphamide, vincristine, doxorubicin, and dexamethasone. The patient was enrolled, his cell were leukapheresed, and he received cyclophosphamide and fludarabine conditioning therapy followed by a single infusion of CD19/CD22 CAR T cells at a dose of 3×10^6^/kg.

Patient 2 was a 24-year-old female with normal cytogenetics. She achieved her CR1 with CDLPD chemotherapy. After multiple cycles of consolidation chemotherapy, this patient subsequently achieved a remission with a negative MRD test. Then, the patient relapsed 13 months later and did not respond to further therapy. The patient was subsequently treated with cyclophosphamide and fludarabine followed by a single infusion of CD19/CD22 CAR T cells at a dose of 2×10^6^/kg.

Patient 3 was a 44-year-old female with relapsed B-ALL (normal cytogenetics). She had received her B-ALL diagnosis 10 years earlier. After diagnosis, she was induced into her CR1 with induction chemotherapy including cytarabine, 6-mercaptopurine and cyclophosphamide. She completed all her consolidation and maintenance treatments but relapsed approximately 38 months after her last maintenance treatment. She had a second remission after reinduction chemotherapy, but the leukemia recurred 37 months later, and she did not have a response to further intensive chemotherapy regimens, including treatments with vincristine, daunorubicin and prednisone. After cyclophosphamide and fludarabine conditioning chemotherapy, bispecific CD19/CD22 CAR T cells were manufactured and infused in a single dose of 2×10^6^/kg.

Patient 4 was a 17-year-old male with B-ALL with normal cytogenetics. After diagnosis, he achieved a remission with a negative MRD test with VDLD chemotherapy but relapsed during maintenance therapy with 6-mercaptopurine and methotrexate. He then received multiple cycles of further intensive chemotherapy without a significant response. He received cyclophosphamide and fludarabine as lymphodepleting chemotherapy regimen before CD19/CD22 CAR T cell infusion.

Patient 5 was a 20-year-old male who received a diagnosis of B-ALL in 2012. He was treated with vincristine, pegaspargase, daunorubicin and dexamethasone which resulted in his CR1. In July 2015, patient 5, in whom blast cells were detected in the cerebrospinal fluid subsequently (CSF), subsequently received intrathecal chemotherapy and radiotherapy, but the central nervous system (CNS) leukemia relapsed 7 months later after the last treatment regimen. Approximately 8 months later, the patient was found to have relapsed disease in the BM. He was then treated with a cyclophosphamide and fludarabine chemotherapy regimen followed by a single infusion of CD19/CD22 CAR T cells at a dose of 2×10^6^/kg.

Patient 6 was a 39-year-old male diagnosed with Ph+ B-ALL who received induction chemotherapy with imatinib , vincristine and prednisone, which induced into his CR1 but the cancer relapsed 6 months later, and he did not have a response despite receiving multiple cytotoxic and biological therapies. He was treated with cyclophosphamide and fludarabine followed by a single infusion of CD19/CD22 CAR T cells on day 1. He was referred for an allogeneic stem cell transplantation (SCT) while in MRD-negative CR and subsequently removed from the study after transplantation.


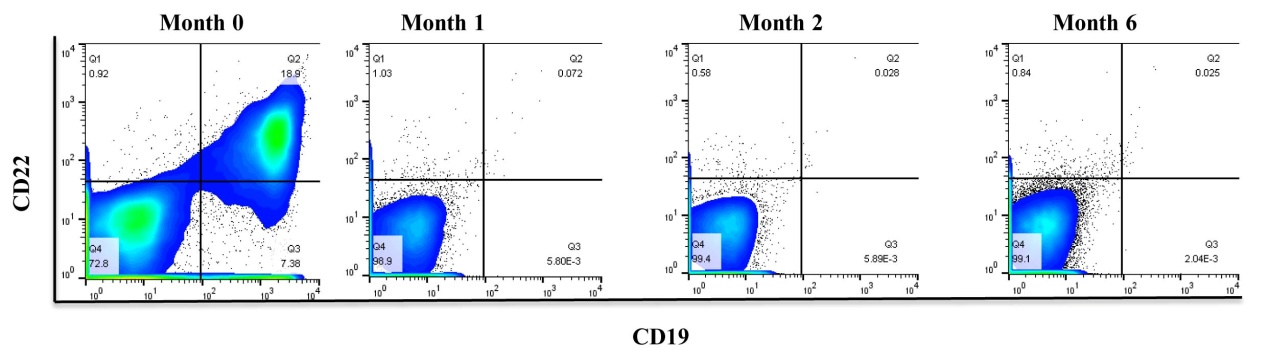


**Figure S1. Persistent B cell aplasia in patient 3**. The panel shows a predominant population of leukemic blast cells in bone marrow spirated from patient 3 expressing CD19 and CD22 on day 0. This population is absent at month 1, month 2, and month 6.

**
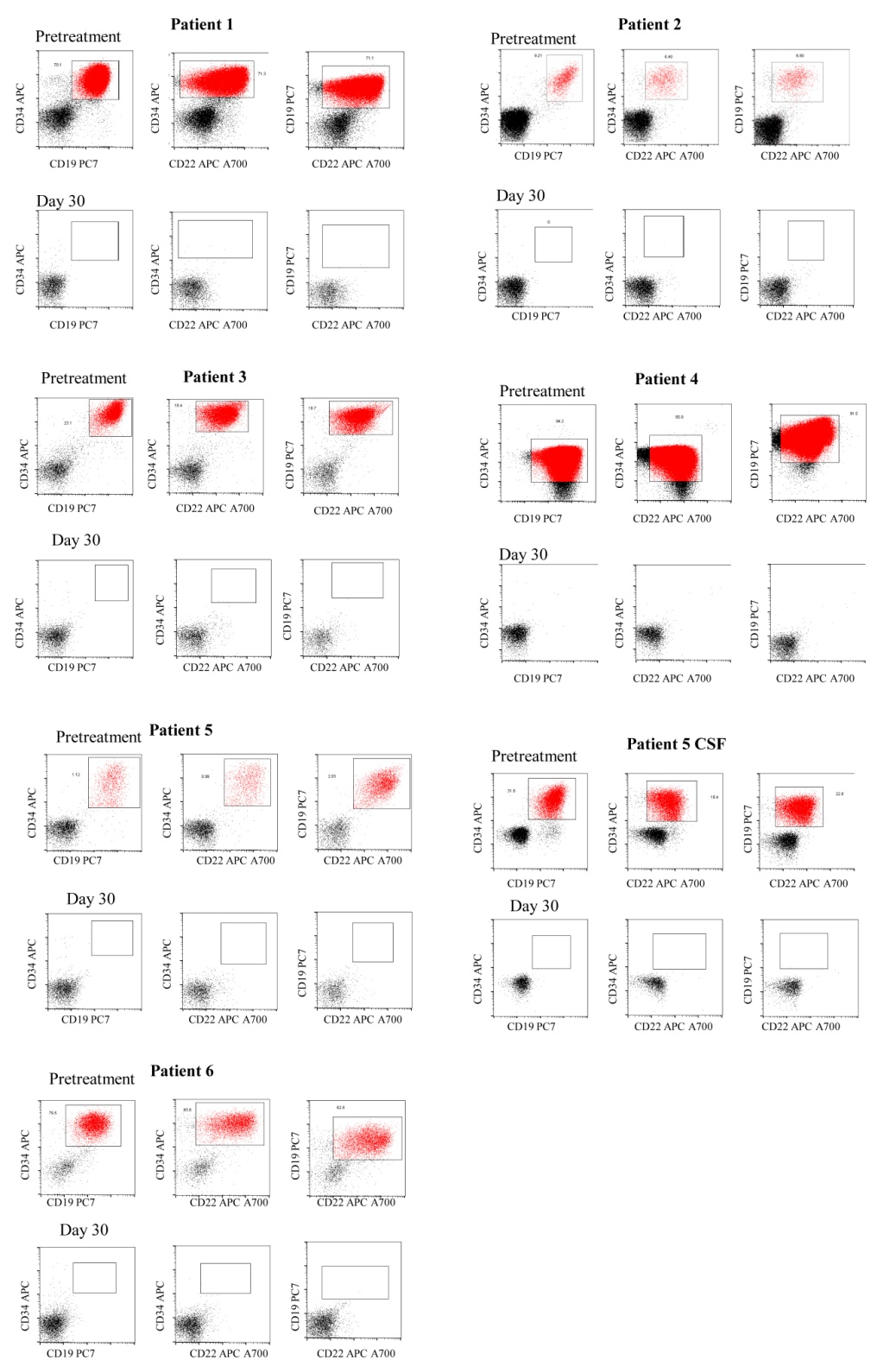
**

**Figure S2. Clinical response to bi-specific CAR T cell therapy.** Eradication of blasts after cell therapy following infusion of CD19/CD22 CAR T cells in all patients.


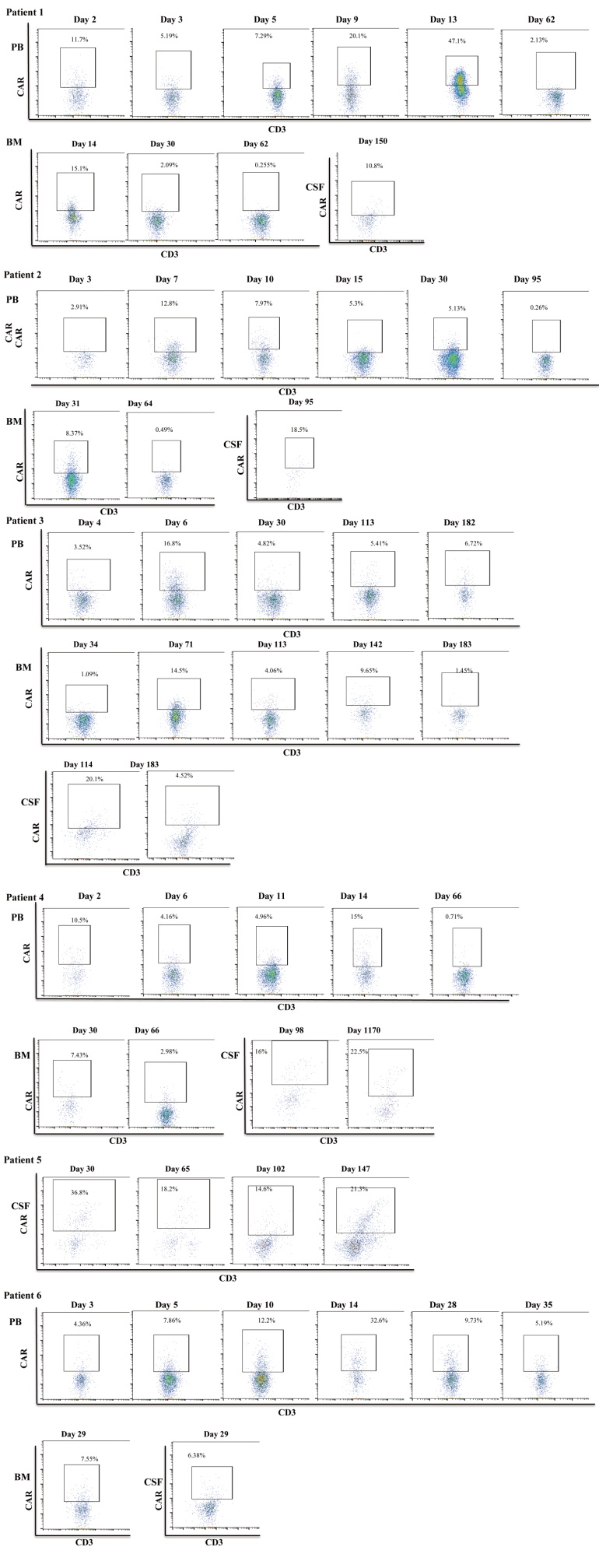


**Figure S3. Persistence and expansion of bispecific CD19/CD22 CAR T cell.** Flow cytometric detection of CD19/CD22 CAR T cells in peripheral blood, BM and CSF from the six patients.


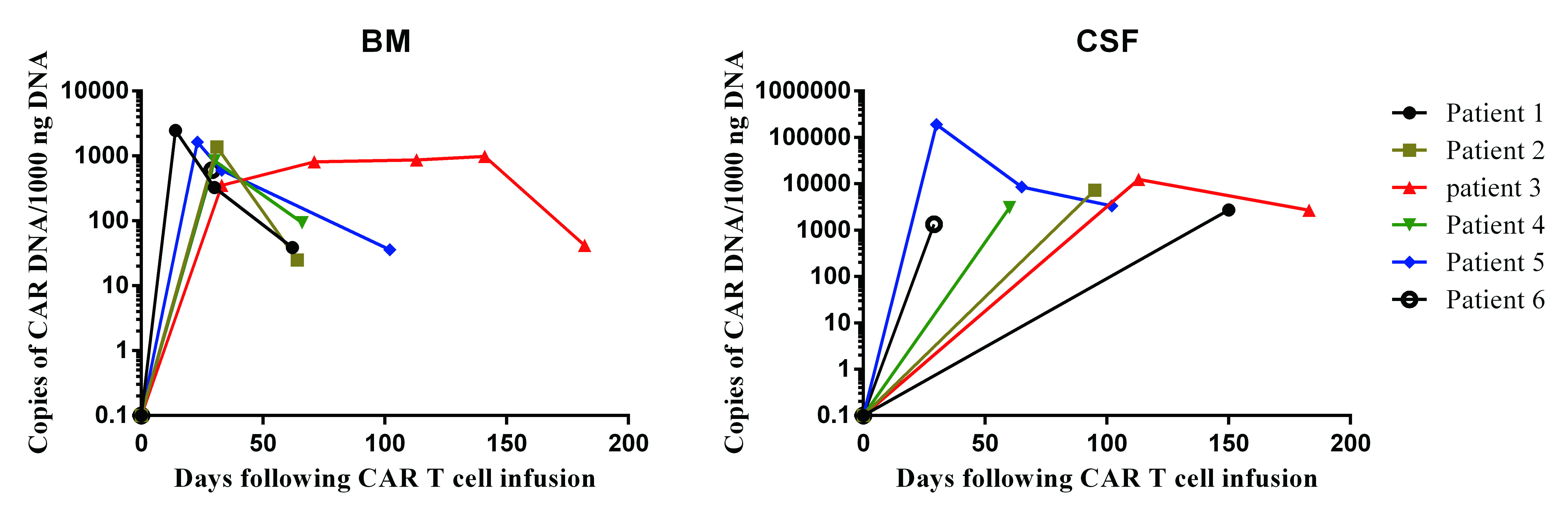


**Figure S4. Bispecific CD19/CD22 CAR T cells in BM and CSF**


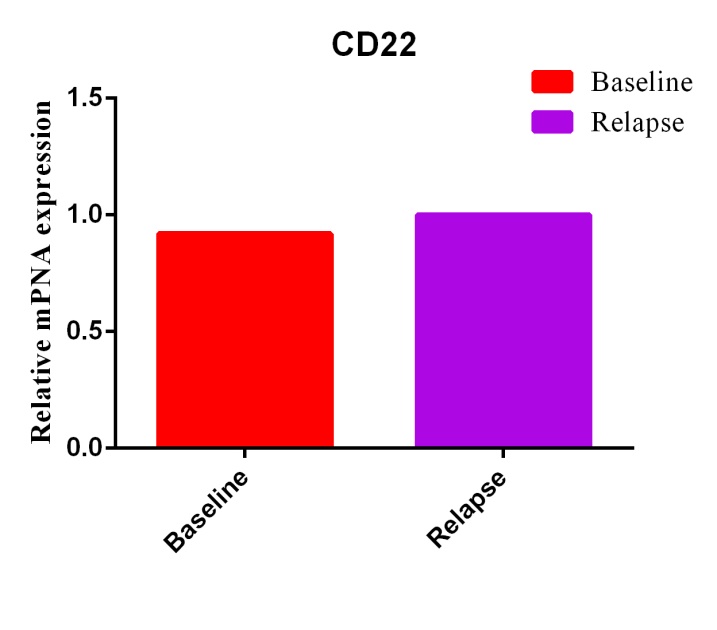


**Figure S5. Analysis of qPCR data for patient 2 showed maintenance of CD22 mRNA expression pre- and post-treatment.**


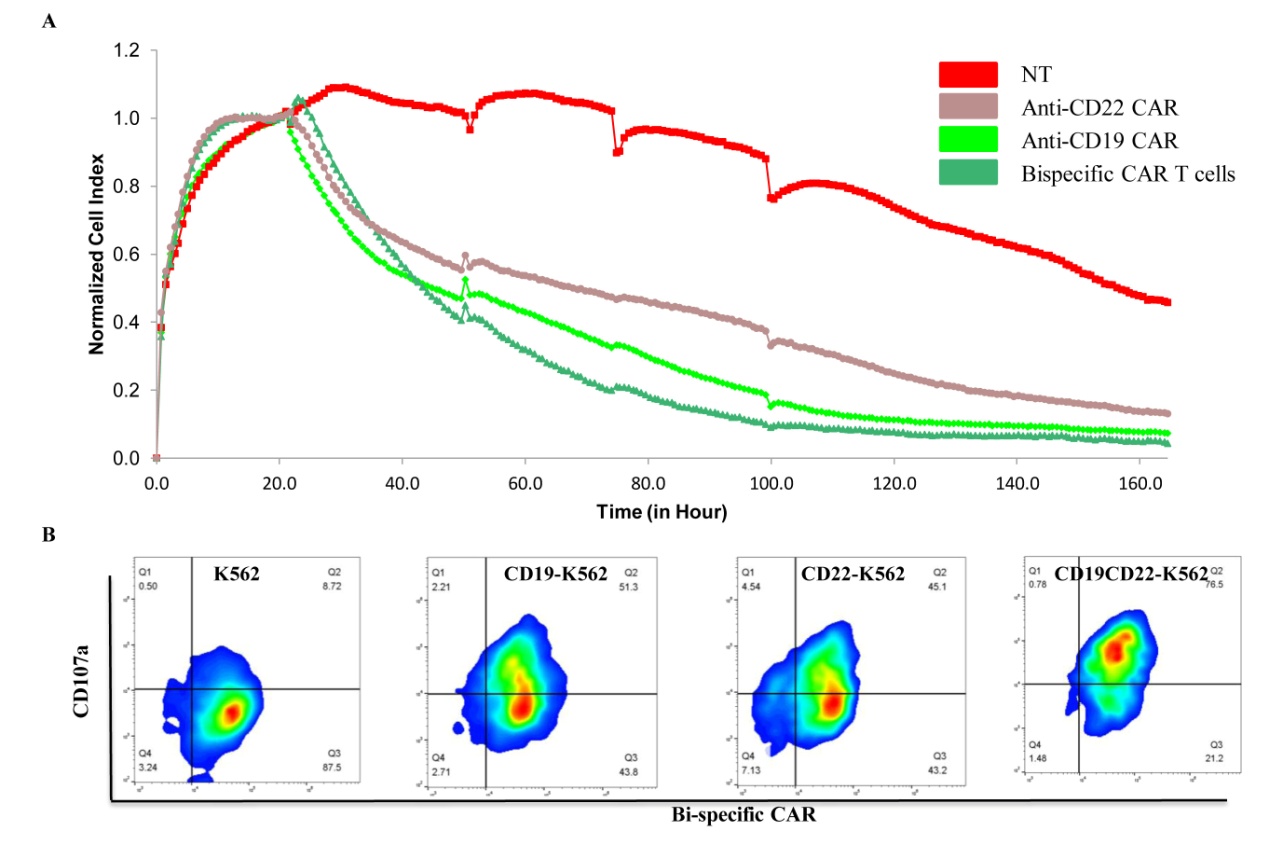


**Figure S6. Functional Bi-specific CAR T cells can be constructed by linking scFv domains in tandem.**

A, Continuous graphical output of cell index values up to the 150-hour time point from Raji during incubation with CART-19, CART-22, and bi-specific CAR T cells and tumor only using the xCELLigence impedance system. Raji cells were seeded in electrodecoated 96-well plates (e-plates) in triplicates. T cells were added at a ratio of 1 T cell for each 1 Raji tumor cells. Electrical impedance was recorded continuously as an indicator of Raji density. B, K562, CD19-K562, CD22-K562 and CD19CD22-K562 cells were cocultured with bi-specific CD19/CD22 CAR T cells for 4 hours. CAR T cells expressed high levels of the degranulation marker CD107a.


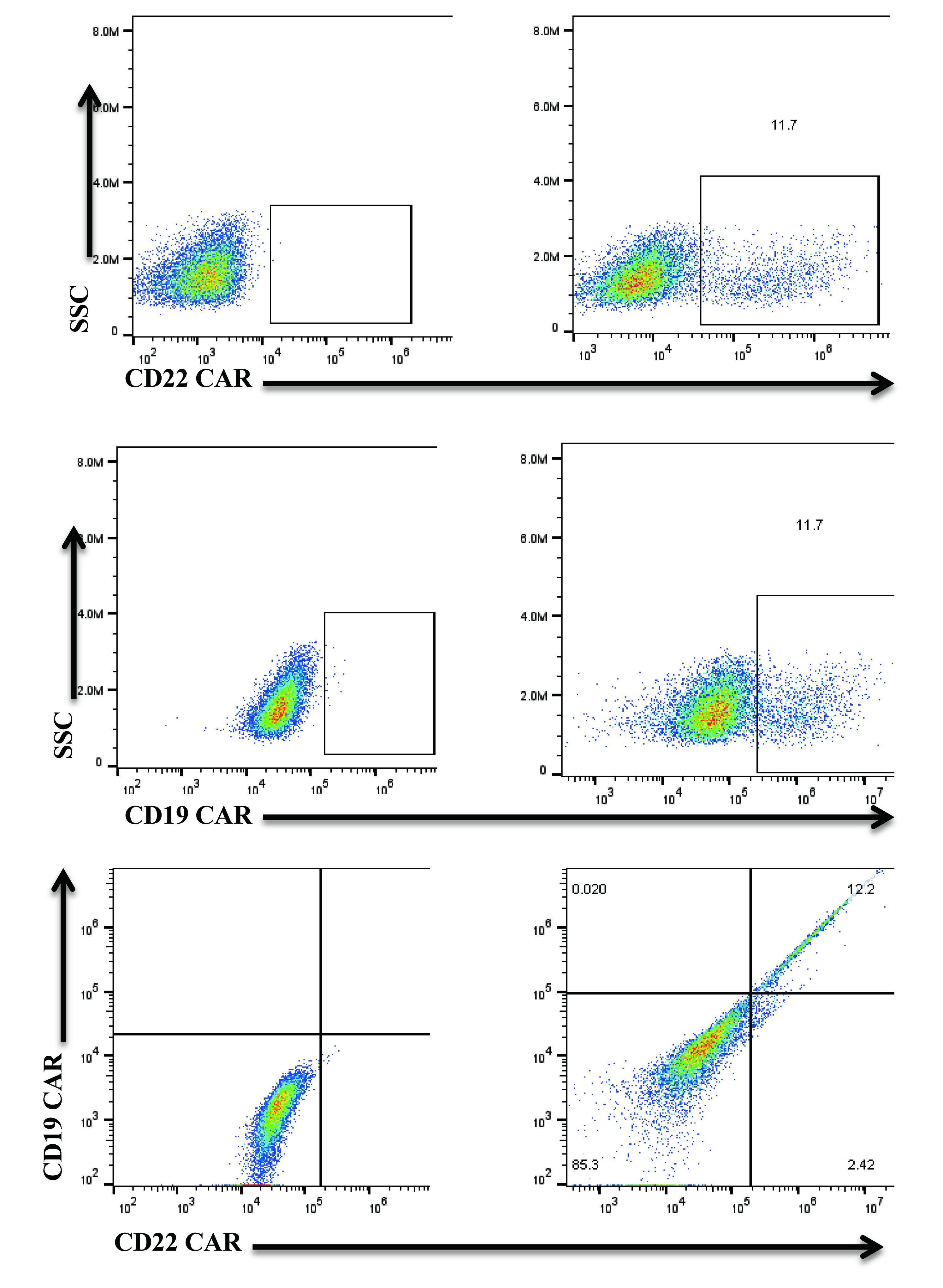


**Figure S7. Flow cytometry analysis of bispecific CD19/CD22 CAR expression in T cells following lentiviral transduction.** Flow-cytometric plot demonstrating the surface binding of CD22Fc and Fab.


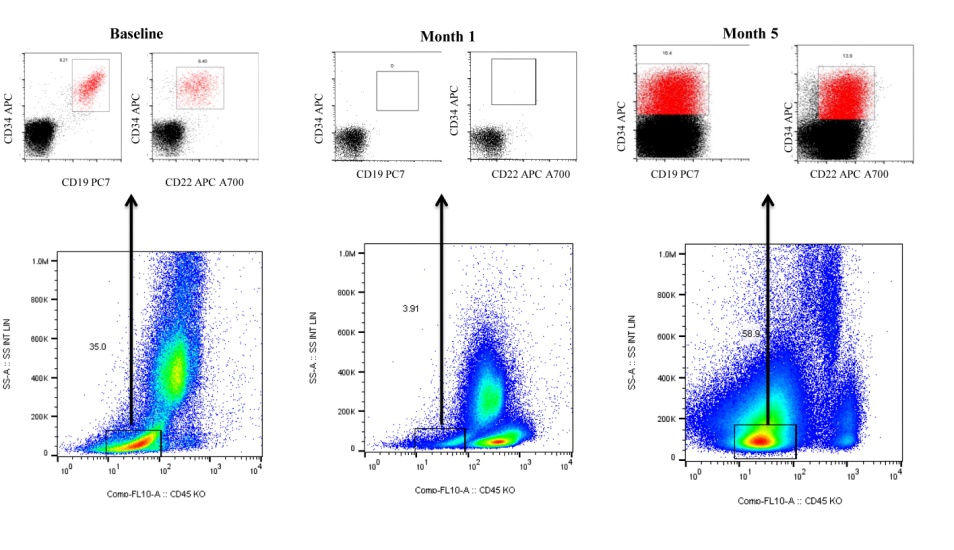


**Figure S8. B cell aplasia and emergence of CD19 and CD22 escape variant cells in patient 2.** Flow cytometric analysis of bone marrow aspirates from patient 2 stained with anti-CD45, CD34,CD19 and CD22. In the bottom row, side scatter and the CD45 dim positive cells were used to identify leukemic cells that express variable amounts of CD34, CD19 and CD22 at baseline. Only CD19 negative and CD22^dim^ blasts were detected on month 5. Numerical values in the top panel represent the fraction of the total leukocytes represented in each quadrant. Numerical values in the lower panel represent the percentage from the total leukocytes represented in the CD45dim/SS low gate.

MALPVTALLLPLALLLHAARPDIQMTQSPSSLSASVGDRVTITCRASQTIWSYLNWYQQRPGKAPNLLIYAASSLQSGVPSRFSGRGSGTDFTLTISSLQAEDFATYYCQQSYSIPQTFGQGTKLEIKRTGGGGSGGGGSGGGGSQVQLQQSGPGLVKPSQTLSLTCAISGDSVSSNSAAWNWIRQSPSRGLEWLGRTYYRSKWYNDYAVSVKSRITINPDTSKNQFSLQLNSVTPEDTAVYYCAREVTGDLEDAFDIWGQGTMVTVSSEAAAKEAAAKEAAAKEVKLQESGPGLVAPSQSLSVTCTVSGVSLPDYGVSWIRQPPRKGLEWLGVIWGSETTYYNSALKSRLTIIKDNSKSQVFLKMNSLQTDDTAIYYCAKHYYYGGSYAMDYWGQGTSVTVSSGGGGSGGGGSGGGGSDIQMTQTTSSLSASLGDRVTISCRASQDISKYLNWYQQKPDGTVKLLIYHTSRLHSGVPSRFSGSGSGTDYSLTISNLEQEDIATYFCQQGNTLPYTFGGGTKLEITDYKDDDDKTRTTTPAPRPPTPAPTIASQPLSLRPEACRPAAGGAVHTRGLDFACDIYIWAPLAGTCGVLLLSLVITLYCKRGRKKLLYIFKQPFMRPVQTTQEEDGCSCRFPEEEEGGCELRVKFSRSADAPAYQQGQNQLYNELNLGRREEYDVLDKRRGRDPEMGGKPRRKNPQEGLYNELQKDKMAEAYSEIGMKGERRRGKGHDGLYQGLSTATKDTYDALHMQALPPR

**Supplemental Figure 9. Sequence of bispecific CAR.**

| Patient | Relapse Day | Nucleotide position (chr16) | Mutation Type | Location of mutation in CD19 | Effect |
| --- | --- | --- | --- | --- | --- |
| 3 | 180 | c.GGGCTGCCAGGCCTGGGAATCCACATGAGGCCCCTGGCCATCTGGCTTTTCATCTTCAACGTCTCTCAACAGATGGGGGGCTTCTACCTGTGCCAGCCGGGGCCCCCCTCTGAGAAGGCCTGGCAGCCTGG>G | DEL | exon 2 | Frame shift (truncating) |

**Supplementary Table 1: Mutations identified by cDNA Sanger sequencing in the CD19 gene for patient 3.** DEL = deletion

| **Patient No.** | **Chemotherapy Dose and Schedule** |
| --- | --- |
|  |  |
| 1 | Flu/Cy |
| 2 | Flu/Cy |
| 3 | Flu/Cy |
| 4 | Flu/Cy |
| 5 | Flu/Cy |
| 6 | Flu/Cy |

**Supplementary Table 2: Lymphodepleting chemotherapy regimens**

Flu/Cy: Fludarabine 30 mg/m^2^ daily ×3 days, Cyclophosphamide 30 mg/kg daily ×2 days

| **Pt.﹟** | **%Transduced** | **%CD3** | **%CD4** | **%CD8** |
| --- | --- | --- | --- | --- |
| 1 | 16.9% | 99.2% | 18.2% | 80.1% |
| 2 | 16.91% | 99.61% | 54.63% | 43.97% |
| 3 | 10.32% | 99.55% | 53.51% | 47.39% |
| 4 | 13.3% | 96.99% | 30.44% | 65.83% |
| 5 | 14.77% | 98.85% | 21.11% | 76.92% |
| 6 | 12.93% | 99.16% | 21.28% | 79.26% |

**Supplementary Table 3: Phenotype of the bispecific CD19/CD22 CAR T cells.**

1. Kochenderfer JN, Dudley ME, Kassim SH, et al. Chemotherapy-refractory diffuse large B-cell lymphoma and indolent B-cell malignancies can be effectively treated with autologous T cells expressing an anti-CD19 chimeric antigen receptor. J Clin Oncol 2015;33:540-9.

2. Dai H, Zhang W, Li X, et al. Tolerance and efficacy of autologous or donor-derived T cells expressing CD19 chimeric antigen receptors in adult B-ALL with extramedullary leukemia. Oncoimmunology 2015;4:e1027469.

3. Kalos M, Levine BL, Porter DL, et al. T cells with chimeric antigen receptors have potent antitumor effects and can establish memory in patients with advanced leukemia. Sci Transl Med 2011;3:95ra73.

4. Shroff A, Sequeira R, Patel V, Reddy KVR. Knockout of autophagy gene, ATG5 in mice vaginal cells abrogates cytokine response and pathogen clearance during vaginal infection of Candida albicans. Cell Immunol 2018;324:59-73.

5. Schwartz A, Marti GE, Poon R, Gratama JW, Fernandez-Repollet E. Standardizing flow cytometry: a classification system of fluorescence standards used for flow cytometry. Cytometry 1998;33:106-14.
